# Role of PPARs in Progression of Anxiety: Literature Analysis and Signaling Pathways Reconstruction

**DOI:** 10.1155/2020/8859017

**Published:** 2020-11-29

**Authors:** Olga I. Rudko, Artemii V. Tretiakov, Elena A. Naumova, Eugene A. Klimov

**Affiliations:** ^1^Faculty of Biology, Lomonosov Moscow State University, Moscow 119234, Russia; ^2^Sirius University of Science and Technology, Sochi 354340, Russia

## Abstract

Peroxisome proliferator-activated receptor (PPAR) group includes three isoforms encoded by PPARG, PPARA, and PPARD genes. High concentrations of PPARs are found in parts of the brain linked to anxiety development, including hippocampus and amygdala. Among three PPAR isoforms, PPARG demonstrates the highest expression in CNS, where it can be found in neurons, astrocytes, and glial cells. Herein, the highest PPARG expression occurs in amygdala. However, little is known considering possible connections between PPARs and anxiety behavior. We reviewed possible connections between PPARs and anxiety. We used the Pathway Studio software (Elsevier). Signal pathways were created according to previously developed algorithms. SNEA was performed in Pathway Studio. Current study revealed 14 PPAR-regulated proteins linked to anxiety. Possible mechanism of PPAR involvement in neuroinflammation protection is proposed. Signal pathway reconstruction and reviewing aimed to reveal possible connection between PPARG and CCK-ergic system was conducted. Said analysis revealed that PPARG-dependent regulation of MME and ACE peptidase expression may affect levels of nonhydrolysed, i.e., active CCK-4. Impairments in PPARG regulation and following MME and ACE peptidase expression impairments in amygdala may be the possible mechanism leading to pathological anxiety development, with brain CCK-4 accumulation being a key link. Literature data analysis and signal pathway reconstruction and reviewing revealed two possible mechanisms of peroxisome proliferator-activated receptors involvement in pathological anxiety: (1) cytokine expression and neuroinflammation mechanism and (2) regulation of peptidases targeted to anxiety-associated neuropeptides, primarily CCK-4, mechanism.

## 1. Introduction

### 1.1. Anxiety and Anxiety Disorders

Anxiety disorders (including generalized anxiety disorders or panic disorders) are the most widespread mental diseases which are at the same time difficult to treat [1, 2]. Main characteristic of panic disorder is presence of repetitive sudden panic attacks [3]. According to large scale surveys, percentage of population suffering from the anxiety disease throughout lifetime is up to 33.7% [4, 5].

Many researchers report that anxiety disorders cause even more severe decrease in patient's quality of life and psychosocial functions than other chronic diseases including diabetes, cardiovascular diseases, and lung diseases [6–8]. Both environmental factors and genetic factors are believed to be involved in the development of panic disorders [9, 10].

Last decade studies showed that anxiety and anxiety disorder are associated with amygdala functioning and various types of its dysfunction that lead to a decrease in its activity [11–15]. Hypothalamus is also often associated with anxiety [16, 17]. Thus, it was shown that the anorexigenic neuropeptide CCK4 is able to directly interact with the hypothalamus [18].

Most modern studies of the molecular basis underlying F40-F48 series (ICD-10) mental disorders focus on polymorphisms in genes encoding the main neurotransmitter system proteins, i.e., catecholamine and GABAergic [19]. However, it is already clear that they are not the primary link in the fine regulation of the formation, severity, and direction of anxious emotions.

### 1.2. Peroxisome Proliferator-Activated Receptors

Peroxisome proliferator-activated receptor (PPAR) subfamily belongs to nuclear receptor family. Three isoforms encoded by individual genes are known: PPAR*γ*, PPAR*α*, and PPAR*δ*. PPARs are ligand-dependent transcription factors that regulate target gene expression by binding to specific peroxisome proliferator response elements (PPREs) in the enhancer sites of regulated genes. Each receptor binds to its PPRE as a heterodimer with a retinoid X receptor (RXR). Upon binding of the agonist, PPAR conformation changes and stabilizes, after which transcriptional coactivators contribute to activation of target genes [20].

The PPARs possess the canonical domain structure common to other nuclear receptor family members, including the amino-terminal AF-1 transactivation domain, followed by a DNA-binding domain and a dimerization and ligand binding domain with a ligand-dependent transactivation function AF-2 located at the carboxy-terminal region [21].

PPARs regulate expression of genes actively involved in lipid and carbohydrate metabolism, vascular homeostasis, tissue repair, cell proliferation and differentiation, and sexual dimorphism [22].

PPARs are expressed in almost all mammalian tissues and organs. The expression patterns of PPAR*α*, PPAR*β*/*δ*, and PPAR*γ* differ, although intersections do occur. A high expression level of all PPARs is observed in tissues with active fatty acid metabolism. PPAR*β*/*δ* is constitutively expressed in almost all tissues [22, 23]. PPAR*γ* among all 3 isoforms has the highest expression in the nervous system, where it was found in neurons, astrocytes, and glial cells [24]. Moreover, all three PPA receptors are expressed both in amygdala and in the hypothalamus [24]. PPAR association with various diseases is also shown [25]. However, the association with anxiety is poorly understood.

This study for the first time analyzes the relationship between PPARs and anxiety. Our results will allow to look at molecular-genetic basis of anxiety disorders pathogenesis, underlying mechanisms and related problems from a new angle.

## 2. Materials and Methods

We used the Pathway Studio® 9 desktop software with ResNet® 14 database and web version of the Pathway Studio software (https://mammalcedfx.pathwaystudio.com) (Elsevier). Additional search of information was performed by using PubMed (http://www.ncbi.nlm.nih.gov/pubmed/), TargetInsights (https://demo.elseviertextmining.com/), and Google Scholar (https://scholar.google.ru/). Signal pathways were created according to previously developed algorithms [26].

**Figure 1:**
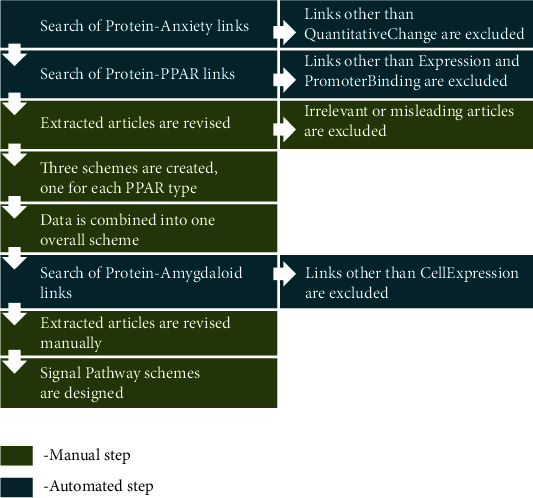
Workflow used in our study. Description in the text.

Search algorithm and workflow scheme are presented in Figure 1. Detailed description is given along in Results for better understanding.

## 3. Results and Discussion

### 3.1. A Search for Common PPARs and Anxiety Targets

During the first step of our work, we used text-mining and signaling pathway analysis for revealing possible role of PPARs in the development of anxiety.

We used the Pathway Studio® 9 desktop software with ResNet® 14 database and web version of the Pathway Studio software (https://mammalcedfx.pathwaystudio.com) (Elsevier).

The search algorithm for anxiety-protein-PPAR links was as follows:

1. Searching proteins linked to anxiety through concentration change. Linkage type: QuantitativeChange was used in Pathway Studio. This type allows to search for proteins with reported concentration alterations in patients with anxiety. Total of 304 proteins were found
2. 304 proteins discovered on the previous step were taken into further analysis by searching linkage between protein and each of the three PPARs independently. We had chosen to search for PPAR connections separately to avoid too complicated pathways. Linkage types: Expression and PromoterBinding were used in Pathway Studio. These two types imply direct PPAR effect on protein concentration through promoter binding or other direct interaction which makes them the most relevant
3. On the next step, all the supporting references were manually revised; at first by reviewing Pathway Studio text-mined “Sentences” section, and after that, if necessary, by studying the whole text for additional details
4. This analysis resulted in three schemes which include all the proteins linked to both anxiety and PPARs. Each scheme was representing one PPAR isoform
5. Three schemes created on the previous step were combined into one for better data representation and reducing number of figures

For describing proteins and their role, search engines highlighted in Materials and Methods were used.

**Figure 2:**
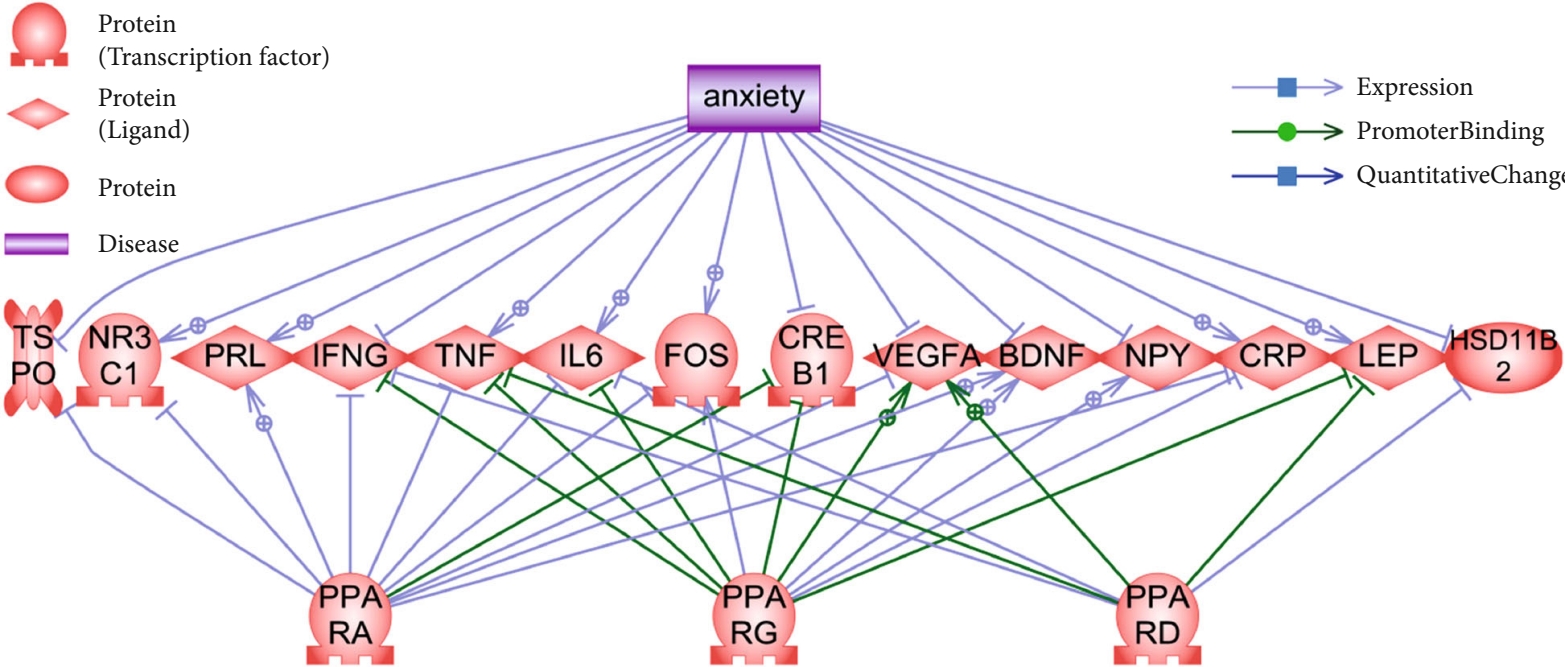
Common targets of anxiety and PPARs. Legend given on the picture.

Thus, lists of proteins with confirmed quantity changes in anxiety, and proteins connected to PPARs were obtained.  Figure 2 and Table 1 show combined target lists.

### 3.2. Common Target Descriptions and Possible Underlying Mechanisms

Only cytokines (IFNG, IL6, and TNF) and vascular endothelial growth factor A (VEGFA) are regulated by all three PPARs. In most cases, connection to PPAR*α* is present. In one case, HSD11B2, only connection to PPARD is present.

Thus, 14 PPAR-regulated proteins with altered concentration in anxiety are known:

#### 3.2.1. BDNF (Brain-Derived Neurotrophic Factor)

Protein's main function is nerve growth and neuron homeostasis [27]. Both BDNF polymorphisms and concentration alterations are linked to variety of mental and neurological diseases [28]. In our case, reduced BDNF product concentration is observed in both animal models ([29], see supplementary (available here)) and patients ([30], see supplementary). The BDNF expression is positively regulated by PPARA ([31], see supplementary) and PPARG ([32], see supplementary). If PPARs-independent BDNF expression regulation pathways are unaltered, lower PPARA and PPARG levels are to be expected as well.

#### 3.2.2. CREB1 (CAMP Responsive Element Binding Protein 1)

This is a transcription factor which induces target gene transcription in response to hormonal stimulation via the cAMP pathway. This protein is involved in many cellular processes and is expressed in all tissues (https://www.ncbi.nlm.nih.gov/gene/1385). CREB1 activity is important for brain neuron development and maintenance [33, 34]. Thus, its linkage to mental diseases' pathogenesis is widely studied [35, 36]. The CREB1 expression is known to be lowered in animal models of anxiety [37, 38]. PPARA and PPARG activate CREB1 transcription by binding to its promoter. Exact in-promoter targets of PPARA are known [39, 40], which points out its important role on CREB1 regulation. PPARG was shown to be able to bind to CREB1 promoter bearing certain nucleotide sequence and block its expression [41]. However, other mechanisms leading to CREB1 activation along with PPARG activation are known, i.e., PKCA (protein kinase C, alpha) activation with further CREB1 activation [42]. Interestingly, CREB1 activation via cAMP-PKCA signaling provokes binding of CREB1 to a cAMP responsive element-like site in PPARG gene promoter region [43] which allows to speculate of mutual regulation of these factors.

#### 3.2.3. CRP (C-Reactive Protein)

This protein is involved in several host defence-related functions based on its ability to recognize foreign pathogens and damaged host cells and initiate their elimination by interacting with humoral and cellular effector systems in the blood (https://www.ncbi.nlm.nih.gov/gene/1401). CRP linkage to anxiety was shown in several studies. Patients with anxiety were shown to have increased CRP levels [44–46], wherein all authors point out the alterations in patients' immune status. This can be explained by possible interactions between the central nervous system and the immune system in neuropsychiatric disorders. CRP regulation via PPARs is negative in both PPARA ([47, 48], see supplementary) and PPARG ([49, 50], see supplementary) cases. There is no information on PPARD-CRP interactions. Thus, in case of anxiety, we can observe an increase of CRP levels despite PPARA and PPARG blocking it. The reasons may lie in either other CRP activation signal pathways or tissue effects insofar CRP is expressed in the liver. This is the only protein in this list which lacks expression brain regions of interest.

#### 3.2.4. FOS

FOS proteins are members of the transcription factor complex AP-1. FOS proteins were implicated as cell proliferation, differentiation, and transformation regulators. FOS gene is expressed in almost every tissue (https://www.ncbi.nlm.nih.gov/gene/2353). One of the most thorough research of this gene is focused on neuronal plasticity [51, 52]. Animal models of anxiety, fear, and depression show increased FOS in the brain areas linked to the formation of these behavioral reactions in animals [53–55]. Negative effect of PPARA activation on the FOS expression is known [56, 57]. It is also known that PPARG regulates the FOS expression [58–61], but there is no univocal opinion wherever this regulation is positive or negative. It is most likely that several signal pathways involving PPARG and FOS are present. Most of the PPARG-FOS interaction studies are using osteogenesis models, which makes it impossible to speculate about PPARs-FOS-anxiety linkage. This linkage is the least evidence-supported among this list.

#### 3.2.5. HSD11B2

The corticosteroid 11-beta-dehydrogenase is a microsomal enzyme complex responsible for the interconversion of glucocorticoid cortisol and its inactive metabolite cortisone; this conversion prevents illicit activation of the mineralocorticoid receptor (https://www.ncbi.nlm.nih.gov/gene/3291). Cortisol is intricately linked to anxiety as increases in its concentration correlates with anxiety and its symptoms [62, 63]. Insofar, as HSD11B2 enzyme is directly involved in processes related to cortisol, its role is pathogenesis of diseases characterized by increased cortisol levels is important [64]. Animal models with HSD11B2 gene knockout have inborn predisposition to increased anxiety [65]. Thus, reduced HSD11B2 activity leads to increased cortisol and anxiety phenotype development. Main organs expressing HSD11B2 are the kidneys, intestines, and salivary glands. Only three of PPAR receptors regulate the HSD11B2 expression wherein this regulation is negative. PPARD is known to bind to HSD11B2 promotor in trophoblasts of the placenta and inhibits its expression [66–68]. We have no knowledge of studies describing PPARD and HSD11B2 interactions in other tissues. However, as far as PPARD is expressed in all tissues, both genes are expressed in the kidney which is the most interesting organ from the point of cortisol accumulation. Thus, in case of HSD11B2, one of peroxisome proliferator-activated receptors (PPARD) may be linked to one of the clinical anxiety manifestations, i.e., increased cortisol.

#### 3.2.6. IFNG

Interferon gamma is soluble cytokine that is a member of the type II interferon class, secreted by cells of both the innate and adaptive immune systems (https://www.ncbi.nlm.nih.gov/gene/3458). Its expression is limited by immune system cells. Interferon gamma may modulate anxiety and depressive states via its role in brain plasticity [69]. Other study suggests that decreased INFG in patients with anxiety disorder is not a cause but a result of the disease [70]. However, all PPARs regulate its expression negatively via INFG promotor binding and blocking its transcriptional activity ([71–75], see supplementary). Thus, link between PPARs and INFG regulation is unequivocal, but determining if decreased INFG concentration is a disease's cause, or a consequence is a subject for further studies. It is possible that decrease of INFG concentration is a result of prolonged anxiety.

#### 3.2.7. IL6 (Interleukin 6)

This cytokine plays role in inflammation and maturation of B cells; encoded protein was shown to be an endogenous pyrogen capable of inducing fever in people with autoimmune diseases or infections (https://www.ncbi.nlm.nih.gov/gene/3569). Increase of the IL6 expression in patients with anxiety/depression is well known [76–80]. However, some studies show decrease of IL6 levels in states characterized by pathological anxiety [81, 82]. Thus, IL6 anxiety link is possible but requires further studies. All three PPARs negatively regulate IL6 ([83–85], see supplementary). Further understanding PPARs and IL6 interaction requires evaluation of IL6 role in anxiety.

#### 3.2.8. LEP

Leptin is secreted by white adipocytes into the circulation and plays a major role in the regulation of energy homeostasis; it binds to the leptin receptor in the brain, which activates downstream signaling pathways that inhibit feeding and promote energy expenditure (https://www.ncbi.nlm.nih.gov/gene/3952). Leptin concentration is increased in patients with emotional anxiety and reaches its peak in conditions of moderate anxiety [86]. Other studies also show high levels of leptin which correlates with anxiety levels [87–89]. Leptin administration resulted in dose-dependent anxiety decrease [86, 90]. This data points out the positive link between leptin and anxiety. Among three PPARs, two receptors regulate LEP gene expression, i.e., delta and gamma. PPARD binds to the LEP promotor and inhibits its expression [91–93]. Thus, one of the mechanisms of leptin increase in anxiety may be linked to PPARD and PPARG decrease.

#### 3.2.9. NPY

Neuropeptide Y is a neuropeptide that is widely expressed in the central nervous system and influences many physiological processes, including cortical excitability, stress response, food intake, circadian rhythms, and cardiovascular function (https://www.ncbi.nlm.nih.gov/gene/4852). Neuropeptide Y deficiency is significantly linked to anxiety development in all animals including fish and human ([94–97], see supplementary). The NPY expression is linked to only one PPAR—gamma; its positive effect on the NPY expression is known in arcuate hypothalamus [98]. We can speculate that NPY decrease may be linked to alterations in PPARG activation/functioning.

#### 3.2.10. NR3C1

Glucocorticoid receptor can function both as a transcription factor that binds to glucocorticoid response elements in the promoters of glucocorticoid responsive genes to activate their transcription and as a regulator of other transcription factors and involved in inflammatory responses, cellular proliferation, and differentiation in target tissues (https://www.ncbi.nlm.nih.gov/gene/2908). Increase of the NR3C1 expression was linked to anxiety increases in rats [99]; antagonist administration resulted in reduction of anxiety-like behavior in rats [100]. Increased glucocorticoid leads to increased anxiety via activation of receptor NR3C1 [101, 102]. Among all peroxisome proliferator-activated receptors, only PPARA is linked to the NR3C1 expression regulation as it reduces it [103–105]. Wherein mutual gene regulation is known as NR3C1 is capable of activating the PPARA expression [106]. Thus, PPARA is capable of regulating NR3C1-dependent anxiety.

#### 3.2.11. PRL

Prolactin is a hormonal growth regulator for many tissues, including cells of the immune system, essential for lactation (https://www.ncbi.nlm.nih.gov/gene/5617). An increase in prolactin levels is associated with anxiety in women during lactation [107], as well as in paratroopers before jumping [108]. In roman low-avoidance rats with an increased level of anxiety, an increased level of the PRL gene expression in the amygdala was shown [109]. PPARA is the only PPAR able of activating the PRL expression [110–112]. It is also noted that gene is not necessarily activated via PPARA binding to PRL promotor [111, 113]. Thus, one of the mechanisms leading to the increased PRL in anxiety may be explained due to PPARA activity.

#### 3.2.12. TNF

Tumor necrosis factor is a multifunctional proinflammatory cytokine that belongs to the tumor necrosis factor superfamily; it is mainly secreted by macrophages and involved in the regulation of a wide spectrum of biological processes including cell proliferation, differentiation, apoptosis, lipid metabolism, and coagulation (https://www.ncbi.nlm.nih.gov/gene/7124). Serum TNF levels are elevated in patients with anxiety symptoms [114], as well as in patients with generalized anxiety disorder [115]. TNF knockout mice showed a low level of anxiety [116]. Cited works indicate a reliable association of TNF elevation with anxiety. All three PPARs block the expression of TNF, as well as the rest of the cytokines identified in our work ([92, 117–121], see supplementary for additional links). Thus, the explanation of increased TNF in anxiety patients lays either in the malfunction/decreased activity of PPARs or in the activation of another mechanism for regulating the TNF expression.

#### 3.2.13. TSPO

Translocator protein is a key factor in the flow of cholesterol into mitochondria to permit the initiation of steroid hormone synthesis and interacts with some benzodiazepines (https://www.ncbi.nlm.nih.gov/gene/706). The level of TSPO was significantly reduced in both patients with anxiety and anxiety mice models [122–125] and increased after treatment [126]. Since the benzodiazepines are used in the treatment of anxiety and anxiety disorder [127], the association of their molecular target TSPO with anxiety is beyond doubt. The TSPO expression is negatively regulated by PPARA ([128, 129], see supplementary). It can be assumed that PPARA activity may adversely affect the development of anxiety due to a decrease in the TSPO expression.

#### 3.2.14. VEGFA (Vascular Endothelial Growth Factor A)

It is a growth factor which induces proliferation and migration of vascular endothelial cells; essential for angiogenesis (https://www.ncbi.nlm.nih.gov/gene/7422). A decrease in VEGFA concentration in patients with anxiety [130, 131] and a high VEGFA level accompanying low anxiety [30] were reported. The VEGFA expression is regulated by all PPARs: delta and gamma bind to the promoter, activating the translation ([132], see supplementary), alpha, on the contrary, blocks VEGFA promoter, reducing expression ([133], see supplementary). Thus, activation and blockade of the VEGFA gene expression are also regulated by transcription factor PPARs. Since the VEGFA expression is reduced in patients with increased anxiety, it can be assumed that in this case, both PPARA activation and a decrease in PPAR delta and gamma activity may occur.  Table 2 summarizes above said.

In our analysis, two groups of proteins are the most presented:

- Inflammatory cytokines—three members (IL6, INFG, and TNF)
- Transcription factors—three members (CREB1, FOS, and NR3C1)

Interestingly, all PPARs are linked to the inflammatory cytokine expression, while only PPARA and PPARG are involved linked to transcription factor expression. Transcriptional targets of PPARD are involved in immune response and body homeostasis (HSD11B2, LEP, and VEGFA). It expresses in all brain tissues at the same level [24], which indicates the probable absence of its participation in the development of most behavioral reactions, including anxiety.

### 3.3. PPARs, Common Target, and Amygdala

Proteins, identified in the previous step, were reviewed for expression in amygdaloid:

1. Amygdaloid was added to the list, and all the amygdaloid-protein links were extracted
2. All the links except for CellExpression type were excluded
3. All the supporting references were manually revised at first by reviewing Pathway Studio text-mined “Sentences” section, and after that, if necessary, by studying the whole text for additional details

**Figure 3:**
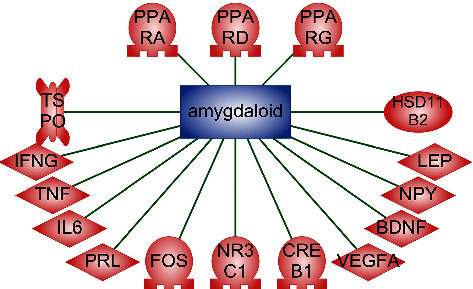
The association of proteins with amygdala. Relation type—CellExpression.

All detected proteins, except for CRP (C-reactive protein), are expressed in amygdala (Figure 3).

Information about the environment is acquired via sensory organs and is transferred to the thalamus nuclei of the limbic system and then to the cortical sections of the sensory analyzer (auditory, visual, tactile cortex):

- The limbic system responds to the image that the brain has perceived and recognized. In particular, amygdala is responsible for the defensive reaction, fear, and aggression
- The amygdala sends a signal to the prefrontal cortex, which evaluates the situation. Its main function in this cascade is to develop a rescue plan in a situation of perceived danger. This path functions inappropriately in case of phobias, which leads to the development of a sense of fear for the stimuli that are not harmful. The amygdala is a key link in the anxiety formation; it is known that the groups of cells of the amygdala are activated when there is fear or aggression. The central nucleus of the amygdala has direct connections with the hypothalamus and brain stem—areas also responsible for fear.

Thus, the amygdala is currently defined as the main part of the brain responsible for the formation of anxiety and fear [13, 134, 135].

PPARs and retinoid X receptor are expressed in the central nervous system. Delta is widely expressed in all parts of the brain, while alpha and gamma show selective expression:

- Gamma is not expressed in the structures of the olfactory bulb, in the part of the olfactory cortex, part of the neocortex, some structures of the thalamus, nuclei of the solitary tract, dorsal motor nuclei of the vagus, and Purkinje cells
- Alpha is not expressed in the hypothalamus [24]

All 3 isoforms are actively expressed in the basal ganglia to which amygdala belongs [24, 136, 137]. Thus, PPARs in high concentration are located in the brain areas involved in the formation of anxiety, including those that are widely represented in the hippocampus and amygdala.

### 3.4. Enrichment Analysis

To further elucidate role of PPARs in anxiety, we conducted Sub-Network Enrichment Analysis (SNEA) using the Pathway Studio® software using list of genes obtained during the first stage of our study. We found considerable number of pathways/gene sets in almost every search subgroup (SNEA: for compounds regulators (branch-drugs), for compounds regulators, for phenotypes and processes (all), with anatomy, with expression regulators) for every gene associated with anxiety disorder.

Table 3 lists top 10 of 100 substances obtained after the SNEA Compounds Regulators Enriched analysis. Compound regulators were chosen as the most representative search subgroup. All these substances (ginsenoside, epinephrine, corticosterone, norepinephrine, Li+, serotonin, n-3 polyunsaturated fatty acid, dehydroepiandrosterone), either have an antistress, antidepressant, neuroprotective, and neurogenic effects or regulate blood pressure, carbohydrate, and lipid metabolism (according to https://pubchem.ncbi.nlm.nih.gov/). Some of them are found in cellular membranes and blood vessel walls. Despite the low Jaccard similarity, which may be explained by relatively low number of searched genes (17), according to enrichment analysis, good ratio of 9/10 of Gene Set Seed related to neurological regulation is observed. This means that 9 substances out of 10 are related to neurological regulation through underlying pathways. This result points out the significant role these genes play in pathogenesis of psychoneurological diseases.

In this set of 10 substances, genes BDNF, FOS, VEGFA, IFNG, IL6, TNF, and PPAR (A, D, G) are present in 100% of underlying pathways. PPARG is present in 80% of them, while PPARA in 70% cases and PPARD in 50%. This tendency is persists in full list of 100 substances obtained after SNEA Compounds Regulators Enriched analysis with BDNF presence in 77%; FOS, 76%; VEGFA, 82%; IFNG, 76%; IL6, 96%; TNF, 96%; and PPAR (A, D, G), 78%. Among PPARs, PPARA is present in 52% and PPARD in 27%; most presented is PPARG in 71%, wherein only 18% out of 100 substances lack any PPARs in their underlying pathway.

This result is an indirect evidence of PPAR role in the anxiety development.

### 3.5. Neuroinflammation, PPARs, and Anxiety

**Figure 4:**
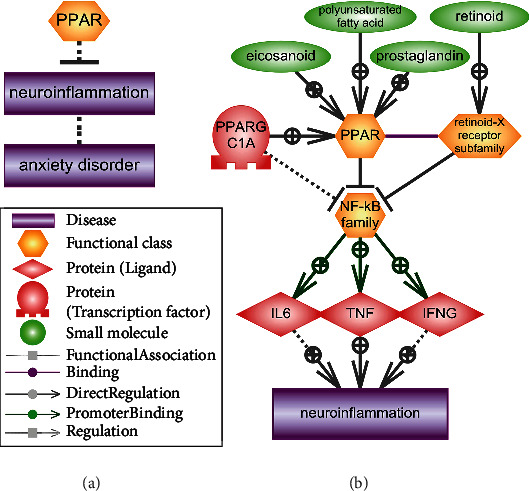
The association of PPARs with neuroinflammation. (a) The effect of PPARs on neuroinflammation and the association of neuroinflammation with anxiety. (b) The signaling pathway of the blockade by activated PPARs of the transcription factor NF-kappa B, an activator of transcription of cytokines IL6, TNF, and IFNG. The legend in the figure.

Recently, the theory of neuroinflammation as the cause of anxiety development was discussed [138–141]. We performed a signal pathway analysis which revealed possible mechanism explaining the role of PPARs in the development of neuroinflammation (Figure 4).

Signal pathway schemes were designed manually using data available in the ResNet database describing links between objects. Annotated schemes available in the Pathway Studio software were used as well.

We suggest that ligand-activated PPARs block the activity of the NF-kappa B family of transcription factors [142, 143]. Coactivators of PPARs-retinoid-X receptor subfamily [144] and PPARG coactivator 1 alpha (PPARGC1A) [145] are also involved in this process. NF-kappa B activates the transcription of number of proinflammatory cytokines, including those that change their expression during anxiety, i.e., IL6 [146], TNF [147], and IFNG [148]. Thus, PPARs normally block the development of neuroinflammation [149, 150].

### 3.6. Peroxisome Proliferator-Activated Receptor Gamma (PPARG) and Cholecystokininergic Systems in Anxiety

In recent years, PPARG was shown to be involved in the development of pathological anxiety [151–153]. This surge of research was initiated by Domi et al., whose aim was to study the role of PPARG in the regulation of GABAergic transmission [154]. The work revealed the role of PPARG in the regulation of mood disorders, indicating that weakened signal transmission can contribute to exacerbation of anxiety and the negative effects of stress. Authors suggest that activation of PPARG may be useful in the treatment of psychiatric conditions related to stress and, in particular, anxiety disorders [154].

At the same time, PPARG is most actively expressed in the amygdala [154–158].

Cholecystokinin (CCK) is a neuropeptide which may be found in high concentrations throughout the central nervous system, where it is involved in numerous physiological functions [159]. The role of CCK, especially its smallest functional peptide, CCK-4, in the induction and maintenance of anxiety and major depression is well known [160–162]. The increase in CCK-4 is associated with loss of motivation, anxiety, and panic attacks [159, 163, 164].

The amygdala is firmly connected with the cholecystokininergic system. It was shown that CCK-4 is synthesized and released into amygdala, hippocampal formation, and cerebral cortex [165, 166]. Both CCK-4 receptors are expressed in the amygdala: CCKAR [167, 168] and CCKBR [168, 169].

In this part of the work, we carried out an analysis of signaling pathways in order to identify a possible connection between the cholecystokininergic system and PPARG.

**Figure 5:**
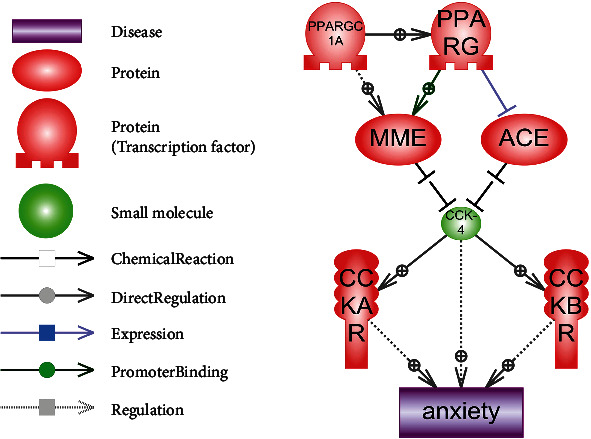
Hypothetical signaling pathway for PPARG in the regulation of anxiety. Legend in the figure.

We used the Pathway Studio® 9 desktop software with the ResNet®14 database and web version of the Pathway Studio software (https://mammalcedfx.pathwaystudio.com) (Elsevier). Signal pathways were created according to the algorithms we developed [170]. The results are presented in Figure 5 and are described below.

PPARG and PPARGC1A are involved in MME transcription regulation [171]. Simultaneously, PPARG inhibits the ACE expression [172].

CCK-4 hydrolysis (Trp-Met-Asp-Phe = WMDF) may be carried out by two enzymes: membrane metalloendopeptidase (MME, also known as NEP, EC 3.4.24.11) and angiotensin I converting enzyme (ACE, EC 3.4.15.1) [173]. MME and ACE cut 2 terminal amino acids (DF) from CCK-4 and CCK-8 which leads to forming of unfunctional peptides (or dipeptides WM and DF in case of CCK-4) [174, 175].

Thus, PPARG-related MME and ACE expression regulation affect levels of active nonhydrolysed CCK-4. Dysregulation of PPARG and the following alteration of peptidase expression in amygdala may be a possible mechanism of pathological anxiety development, with a CCK-4 accumulation as a main cause. This may explain Domi et al. results [154].

## 4. Conclusions

We examined possible associations of PPARs with anxiety. An analysis of the data and signaling pathways available in the literature suggests two mechanisms for the participation of peroxisome proliferator-activated receptors in the formation of anxiety: (1) expression of cytokines and neuroinflammation and (2) regulation of the expression of peptidases, the targets of which are neuropeptides associated with anxiety (CCK-4 in the first place). Further research in these areas will help to better understand the role of PPARs in the development of anxiety.

## Figures and Tables

**Table 1:** List of 14 anxiety-associated proteins–targets of PPARs.

Protein	Full name	Functional class	PPARs
BDNF	Brain derived neurotrophic factor	Growth factor	PPARA PPARG

CREB1	cAMP responsive element binding protein 1	Class bZIP transcription factor	PPARA PPARG

CRP	C-reactive protein	Plasma protein	PPARA PPARG

FOS	FOS proto-oncogene, AP-1 transcription factor subunit	Class bZIP transcription factor	PPARA PPARG

HSD11B2	Hydroxysteroid 11-beta dehydrogenase 2	Steroid metabolism protein	PPARD

IFNG	Interferon gamma	Inflammatory cytokine	PPARA PPARD PPARG

IL6	Interleukin 6	Inflammatory cytokine	PPARA PPARD PPARG

LEP	Leptin	Adipokine	PPARD PPARG

NPY	Neuropeptide Y	Neuropeptide	PPARG

NR3C1	Nuclear receptor subfamily 3 group C member 1	Nuclear steroid hormone receptor family	PPARA

PRL	Prolactin	Glycopeptide hormone (gonadotropins)	PPARA

TNF	Tumor necrosis factor	Inflammatory cytokine/adipokine	PPARA PPARD PPARG

TSPO	Translocator protein	Mitochondrial damage protein	PPARA

VEGFA	Vascular endothelial growth factor A	Growth factor	PPARA PPARD PPARG

**Table 2:** Anxiety-associated proteins and PPARs—directions of regulation.

Target	PPARA	PPARD	PPARG
*BDNF*	**↑**		**↑**
*CREB1*	**↓**		↓
*CRP*	↓		↓
**FOS**	↓		│
*HSD11B2*		↓	
*IFNG*	↓	↓	↓
**IL6**	↓	↓	↓
**LEP**		↓	↓
*NPY*			↑
**NR3C1**	↓		
**PRL**	↑		
**TNF**	↓	↓	↓
*TSPO*	↓		
*VEGFA*	↓	↑	↑

↑—positive regulation (increased expression), ↓—negative regulation (decreased expression), │—exact effect it unclear, empty field—interaction of this target gene with PPAR is unknown. Italics—decreased expression in anxiety, bold—increased expression in anxiety.

**Table 3:** Top 10 for compound regulators subnetworks enriched for anxiety disorder-associated genes.

Gene Set Seed	Total number of neighbours	Overlap	Percent overlap	*P* value	Jaccard similarity
Ginsenoside	106	11	10	1.97889*E*-14	0.098214
Alpha-MSH	179	12	6	1.50466*E*-13	0.065217
Epinephrine	292	13	4	1.50691*E*-12	0.043919
Corticosterone	510	15	2	2.00908*E*-12	0.029297
Norepinephrine	412	14	3	3.86353*E*-12	0.033735
Li+	418	14	3	4.72452*E*-12	0.033254
Serotonin	239	12	5	4.9582*E*-12	0.04918
n-3 Polyunsaturated fatty acid	426	14	3	6.14958*E*-12	0.032634
Dehydroepiandrosterone	330	13	3	7.35581*E*-12	0.038922

*P* value was calculated using Fisher's exact test. Enrichment analysis that does not include experimental values when calculating enrichment from a list. The Jaccard similarity index (Jaccard similarity coefficient) compares members for two sets to see which members are shared and which are distinct. It is a measure of similarity for the two sets of data, with a range from 0% to 100%.

## Data Availability

All supplemental materials (Excel file) are available to download from ResearchGate resource by the link https://www.researchgate.net/publication/340418909_Supplemental_Materials_Role_of_PPARs_in_progression_of_anxiety. Additional data is available from the corresponding author upon reasonable request.

## Supplementary Materials

Supplementary Materials The supplement file was provided for this study (an excel file), including following information: PPARs and neuroinflammation relation sheet, PPARs and CCK4 relation sheet, PPARs and anxiety common target sheet, and amygdaloid expressed gene sheet. The data on relations between objects on pathways. The downloadable online version can be accessed via ResearchGate: https://www.researchgate.net/publication/340418909_Supplemental_Materials_Role_of_PPARs_in_progression_of_anxiety.
