# Supplementary material for: Role of PPARs in Progression of Anxiety: Literature Analysis and Signaling Pathways Reconstruction
Source: PPAR Res. 2020 Nov 29;2020:8859017. doi: 10.1155/2020/8859017 (PMC7721491; doi:10.1155/2020/8859017)
Supplement: Supplementary Materials — The supplement file was provided for this study (an excel file), including following information: PPARs and neuroinflammation relation sheet, PPARs and CCK4 relation sheet, PPARs and anxiety common target sheet, and amygdaloid expressed gene sheet. The data on relations between objects on pathways. The downloadable online version can be accessed via ResearchGate: https://www.researchgate.net/publication/340418909_Supplemental_Materials_Role_of_PPARs_in_progression_of_anxiety. [file 8859017.f1.docx]

| Relation | Type | # of References | Effect | PMID | Tissue | CellType | | Organ |
| --- | --- | --- | --- | --- | --- | --- | --- | --- |
| IFNG --+> neuroinflammation | Regulation | 18 | positive | 22100227, 18852291, 30065723, 29061029, 25927213, 16781017, 23177721, 26797042, 23523267, 23333393 |  | microglia {CellType urn:agi-ncimcelltype:C0206116}, effector T-cell {CellType urn:agi-ncimcelltype:CL439077}, astrocyte {CellType urn:agi-ncimcelltype:C0004112}, helper T-cell {CellType urn:agi-ncimcelltype:C0018894}, neuron {CellType urn:agi-ncimcelltype:C0027882}, B-lymphoblast {CellType urn:agi-ncimcelltype:C1516097}, microglia, T-cell, natural killer cell, neuron | | central nervous system {Organ urn:agi-ncimorgan:C0927232}, brain {Organ urn:agi-ncimorgan:C1269537}, central nervous system, lymph node of mesentery, nervous system, brain |
| IL6 --+> neuroinflammation | Regulation | 86 | positive | 26196302, 18068740, 24088994, 21678079, 27049383, 23840908, 29733875, 27561705, 27443846, 25896970 <more data available...> | muscle {Tissue urn:agi-ncimtissue:C0026845}, plasma {Tissue urn:agi-ncimtissue:C1609077}, muscle, plasma, skeletal muscle, peripheral tissue, parenchyma | endothelial cell {CellType urn:agi-ncimcelltype:C0225336}, keratinocyte {CellType urn:agi-ncimcelltype:C0022567}, astrocyte {CellType urn:agi-ncimcelltype:C0004112}, astrocyte, phagocyte, macrophage, neuron, endothelial cell, neuroglia, T-cell <more data available...> | | retina {Organ urn:agi-ncimorgan:C1962966}, brain {Organ urn:agi-ncimorgan:C1269537}, central nervous system {Organ urn:agi-ncimorgan:C0927232}, blood vessel {Organ urn:agi-ncimorgan:C0005847}, nervous system {Organ urn:agi-ncimorgan:C0027763}, brain, fetal brain, central nervous system, peripheral nervous system, hippocampus <more data available...> |
| TNF --+> neuroinflammation | Regulation | 87 | positive | 17662717, 18455351, 20206673, 19800810, 28242160, 18359564, 29518527, 30196020, 17475413, 29247683 <more data available...> | white matter, peripheral tissue | mesothelial cell {CellType urn:agi-ncimcelltype:C0225335}, neuron {CellType urn:agi-ncimcelltype:C0027882}, brain cell {CellType urn:agi-celltype:brain%20cell}, retinal ganglion cell {CellType urn:agi-ncimcelltype:C0035316}, astrocyte {CellType urn:agi-ncimcelltype:C0004112}, mesothelial cell, immunocompetent cell, neuron, microglia, retinal ganglion cell <more data available...> | | spinal cord {Organ urn:agi-ncimorgan:C0037925}, peritoneum {Organ urn:agi-ncimorgan:C0496954}, central nervous system {Organ urn:agi-ncimorgan:C0927232}, brain {Organ urn:agi-ncimorgan:C1269537}, spinal cord, peritoneum, paw, cerebral cortex, central nervous system, hippocampal CA1 region <more data available...> |
| PPAR ---- retinoid-X receptor subfamily | Binding | 115 |  | 27470447, 28267642, 17632107, 28774777, 15219816, 29510153, 21354188, 17307062, 16288935, 29502862 <more data available...> | bile, adipose tissue, skeletal muscle, kidney parenchyma, plasma, brown adipose tissue, endothelium | adipocyte {CellType urn:agi-ncimcelltype:C0206131}, cardiac myocyte {CellType urn:agi-ncimcelltype:C0225828}, astrocyte, hepatic stellate cell, cardiac myocyte, hepatocyte, stromal cell, adipocyte, oligodendroglia, kidney mesangial cell <more data available...> | | liver {Organ urn:agi-ncimorgan:C0023884}, brain {Organ urn:agi-ncimorgan:C1269537}, artery wall {Organ urn:agi-ncimorgan:C0507850}, placenta, brain, artery wall, liver, uterus, intestine, colon <more data available...> |
| PPARGC1A ---\| NF-kB family | Regulation | 16 | negative | 24327607, 26159922, 20404331, 23770291, 27255468, 23000245, 27084848, 26453501, 21927016, 28268115 | skeletal muscle {Tissue urn:agi-ncimtissue:C0242692}, muscle {Tissue urn:agi-ncimtissue:C0026845}, white adipose tissue | muscle cell {CellType urn:agi-ncimcelltype:C0596981}, insulin-secreting cell {CellType urn:agi-ncimcelltype:C0030281}, microglia {CellType urn:agi-ncimcelltype:C0206116}, skeletal muscle cell, muscle cell | | brain |
| retinoid --+> retinoid-X receptor subfamily | DirectRegulation | 20 | positive | 14522385, 14649582, 16203149, 15281009, 19464395, 18755362, 10218817, 21034986, 12167430, 15375804 <more data available...> | adipose tissue {Tissue urn:agi-ncimtissue:C0001527}, epithelium {Tissue urn:agi-ncimtissue:C0014609}, epidermis {Tissue urn:agi-ncimtissue:C0014520}, adipose tissue, epithelium, epidermis |  | | lung {Organ urn:agi-ncimorgan:C1278908}, head and neck {Organ urn:agi-ncimorgan:C0460004}, lung |
| NF-kB family --+> TNF | PromoterBinding | 30 | positive | 31545910, 31581558, 22151742, 30028921, 10921504, 28822039, 31284759, 12932353, 17679555, 12061770 <more data available...> | | macrophage {CellType urn:agi-ncimcelltype:C1550652}, M2 macrophage {CellType urn:agi-celltype:m2%20macrophage}, mast cell {CellType urn:agi-ncimcelltype:C0024880}, neutrophil {CellType urn:agi-ncimcelltype:C0027950}, macrophage, monocyte | | kidney medulla {Organ urn:agi-ncimorgan:C0022664} |
| NF-kB family --+> IL6 | PromoterBinding | 333 | positive | 19479862, 27539364, 12219016, 30283098, 30028921, 11431008, 30952011, 20939807, 18639630, 30794283 <more data available...> | serum, epithelium, myocardium, adipose tissue, skeletal muscle, Muscle, Smooth, Vascular, blood, endothelium | fibroblast {CellType urn:agi-ncimcelltype:C1550637}, capillary endothelial cell {CellType urn:agi-ncimcelltype:C1180238}, enterocyte {CellType urn:agi-ncimcelltype:C0682610}, umbilical vein endothelial cell {CellType urn:agi-ncimcelltype:C3179121}, B-cell {CellType urn:agi-ncimcelltype:C0004561}, neutrophil {CellType urn:agi-ncimcelltype:C0027950}, pancreatic stellate cell {CellType urn:agi-ncimcelltype:C2936598}, aortic smooth muscle cell {CellType urn:agi-celltype:aortic%20smooth%20muscle%20cell}, endothelial cell <more data available...> | | skin {Organ urn:agi-ncimorgan:C0684084}, lung {Organ urn:agi-ncimorgan:C1278908}, brain {Organ urn:agi-ncimorgan:C1269537}, neurosecretory system, synovial membrane, retina, lung, bone marrow, hypothalamus, liver <more data available...> |
| NF-kB family --+> IFNG | PromoterBinding | 10 | positive | 19160541, 9374532, 12759422, 9797468, 12842891, 16319921, 10227974, 12841650, 8982782, 20421878 |  |  |  | |
| retinoid-X receptor subfamily ---\| NF-kB family | DirectRegulation | 11 | negative |  |  | Kuppfer cell, macrophage, B-cell |  | |
| polyunsaturated fatty acid --+> PPAR | DirectRegulation | 11 | positive |  | plasma, skeletal muscle | lymphocyte, neuron, insulin-secreting cell | | liver, skin |
| eicosanoid --+> PPAR | DirectRegulation | 11 | positive |  |  | monocyte | | liver, central nervous system |
| PPAR ---\| NF-kB family | DirectRegulation | 11 | negative |  | myotube, white adipose tissue | endothelial cell, helper T-cell, macrophage, brain cell, inflammatory cell | | liver, kidney |
| PPARGC1A --+> PPAR | DirectRegulation | 11 | positive |  | plasma | brown adipocyte, cardiac myocyte, muscle cell, neuron | | liver, heart, respiratory system |
| prostaglandin --+> PPAR | DirectRegulation | 11 | positive |  | glomerular mesangium, epithelium | immunocompetent cell | | uterus, brain, intestine |
| PPAR ---\| neuroinflammation | Regulation | 11 | negative | 19607969, 20138762, 24424383, 30836921, 25246934, 28751020, 19698765 |  | neuron {CellType urn:agi-ncimcelltype:C0027882}, endothelial cell {CellType urn:agi-ncimcelltype:C0225336}, cholinergic nerve cell {CellType urn:agi-celltype:cholinergic%20nerve%20cell}, neural stem cell {CellType urn:agi-ncimcelltype:CL016117}, dopaminergic neuron {CellType urn:agi-ncimcelltype:C1512035}, neuron | | nervous system {Organ urn:agi-ncimorgan:C0027763}, head {Organ urn:agi-ncimorgan:C1281590}, hippocampus {Organ urn:agi-ncimorgan:C0019564}, nervous system |
